# Meta-analysis of RAAS inhibition in patients with a systemic right ventricle

**DOI:** 10.1016/j.ijcchd.2026.100705

**Published:** 2026-08-13

**Authors:** Yousef Baroudi, Bengt Johansson

**Affiliations:** Department of Diagnostics and Intervention, Umeå University, Umeå, Sweden

**Keywords:** ACHD, sRV, RAAS-Blockade, Meta-analysis, TGA

## Abstract

**Introduction:**

Pharmacological therapy has transformed the management of heart failure, yet pivotal trials have primarily focused on acquired left ventricular disease. Evidence for renin-angiotensin-aldosterone system (RAAS) inhibition in systemic right ventricle (sRV) remains scarce, and current practice is largely based on extrapolation from left ventricular studies and expert consensus. This meta-analysis evaluates the efficacy and safety of RAAS blockade in patients with a failing sRV.

**Methods:**

A PubMed search identified studies investigating RAAS inhibition in sRV failure. Reported outcomes included changes in NT-pro-BNP, right ventricular ejection fraction (RVEF), and peak oxygen uptake (VO_2_-max). Data were analyzed using conventional statistics and visualized with forest plots.

**Results:**

Ten eligible studies were included, comprising a pooled cohort of 325 patients, of which 261 received active treatment. The population was predominantly male (67.1%), with mean cohort ages ranging from 25.2 to 48.5 years. Mean treatment durations spanned 3.5 to 37.2 months. RAAS inhibition was associated with a reduction in NT-pro-BNP (Cohen's d = –0.28, 95% CI –0.49 to –0.07, *p* = 0.01). However, no improvements in RVEF or VO_2_-max were observed. The therapy was generally well-tolerated. The overall mortality incidence was 5.6 deaths per 1000 person-years in the active treatment groups.

**Conclusion:**

The evidence for RAAS inhibition in patients with sRV is limited but suggests a beneficial reduction in NT-pro-BNP, indicating a hemodynamic effect but without improvements in RVEF or VO_2_-max. When combined with extrapolated data from large left ventricular heart failure trials, these findings support that RAAS inhibition is safe and may be considered in patients with sRV failure.

## 1 Introduction

Congenital heart disease (CHD) remains one of the most common birth defects and a leading cause of mortality among congenital abnormalities [1,2]. Remarkable advances in prenatal diagnostics, transcatheter interventions, cardiothoracic surgery, and perioperative intensive care have dramatically improved survival rates over recent decades. Today, CHD affects approximately 1% of newborns, and in high-income countries, roughly 97% of these individuals survive into adulthood [3,4]. Consequently, the population of adults with CHD (ACHD) is rapidly expanding, with patients reaching unprecedented ages; currently, adults outnumber children within the CHD population [5]. While survival has improved across all phenotypes, the management of this growing cohort of patients with complex CHD poses a significant and ongoing challenge in adult cardiology.

An intricate subset of complex CHD involves a systemic right ventricle (sRV), a condition where the morphologically right ventricle acts as the primary pump supporting the systemic circulation. This anatomy primarily arises from two clinical scenarios. The first is d-transposition of the great arteries (d-TGA) treated historically with an atrial switch procedure (Mustard or Senning operation) prior to the widespread adoption of the arterial switch operation (ASO). The second scenario is congenitally corrected transposition of the great arteries (ccTGA), characterized by atrioventricular and ventriculoarterial discordance, where the right atrium empties into a morphological left ventricle pumping to the pulmonary artery, and the left atrium connects to a morphological right ventricle pumping into the aorta [6].

Because the right ventricle is structurally unsuited to sustain systemic vascular resistance over the long term, progressive heart failure (HF) is a frequent and severe complication [7]. However, the optimal pharmacological management of sRV failure remains controversial, and current practice is largely based on expert consensus and extrapolation from left ventricular studies [[7], [8], [9]]. The 2021 European Society of Cardiology (ESC) guidelines for acute and chronic heart failure emphasize that evidence regarding ACHD management is scarce and recommend that these patients be managed in specialized expert centers [7]. While these guidelines note that sacubitril/valsartan might reduce morbidity, no formal recommendation can be made. Similarly, the 2020 ESC guidelines for ACHD explicitly state that while diuretics are indicated for symptom relief, no solid recommendations can be given for traditional neurohormonal therapies such as angiotensin converting enzyme inhibitors (ACEis), angiotensin receptor blockers (ARBs), beta-blockers, or aldosterone antagonists due to a lack of robust outcome data [8]. The ESC finally underscores that directly translating results from standard left-ventricular heart failure trials to the sRV population is fundamentally inappropriate [10].

Parallel to the European perspective, the American Heart Association (AHA) and American College of Cardiology (ACC) note that randomized controlled trials, retrospective studies, and previous meta-analyses have largely failed to demonstrate a clear clinical benefit for traditional neurohormonal therapies or phosphodiesterase-5 inhibitors across the spectrum of sRV patients [11]. Although newer heart failure agents, such as sacubitril/valsartan and sodium-glucose cotransporter-2 (SGLT2) inhibitors, have demonstrated safety and potential efficacy in non-randomized cohorts, definitive evidence remains sparse [11]. Conversely, some recent observational data, such as that by Woudstra et al., suggest potential prognostic benefits of renin-angiotensin-aldosterone system (RAAS) inhibition and betablockers in symptomatic d-TGA patients with severe sRV systolic dysfunction [12].

This collective evidence highlights a knowledge gap and a lack of international consensus regarding the pharmacotherapy of sRV failure. While a meta-analysis evaluating medical therapy in sRV was conducted in 2018 [13], several clinical studies [[14], [15], [16], [17]] and registry data have emerged since then, offering fresh insights. To address this evolving landscape and clarify the clinical efficacy of neurohormonal blockade, we conducted an updated meta-analysis to evaluate the effect of RAAS inhibition on clinical outcomes and ventricular function in patients with a systemic right ventricle.

## 2 Methods

### 2.1 Search strategy and selection criteria

A systematic literature search on PubMed was conducted from inception up to June 1, 2026, to identify clinical studies evaluating RAAS blockade in adult patients with sRV. The search strategy utilized combinations of Medical Subject Headings (MeSH) and free-text terms, including: “systemic right ventricle”, “transposition of the great arteries”, “atrial switch”, “Mustard”, “Senning”, “congenitally corrected transposition”, “angiotensin-converting enzyme inhibitors”, “angiotensin receptor blockers”, “mineralocorticoid receptor antagonists”, and “sacubitril/valsartan”. The complete search strategy is provided in Supplementary files.

Studies were eligible for inclusion if they met the following criteria:

• Included adult patients (≥18 years of age) with a biventricular circulation and a morphologic sRV (either d-TGA post-atrial switch or ccTGA).‬‬‬‬‬‬‬‬‬‬‬‬‬‬‬‬‬‬‬‬‬
• Evaluated systemic pharmacological therapy using ACE-inhibitors (ACEi), angiotensin receptor blockers (ARBs), mineralocorticoid receptor antagonists (MRAs), or angiotensin receptor-neprilysin inhibitors (ARNIs).
• Reported quantitative data regarding baseline demographics, safety/tolerability (adverse events, treatment discontinuations), or clinical efficacy endpoints (NT-pro-BNP, VO_2_-max, or RVEF).

### 2.2 Statistical analysis and data transformation

All calculations were performed using SPSS version 29.0.2.0 (IBM, Armonk, NY, USA). Categorical variables were summarized as counts and percentages (n, %). Continuous variables were expressed as mean ±‬ standard deviation (SD). For studies that reported continuous baseline demographics or outcome variables as median and interquartile range (IQR), mean and SD values were mathematically estimated using the transformation method described by Wan et al. [18]. Clinical outcomes were calculated using Random-effects model and visually evaluated using forest plots. The null hypothesis was rejected for *p*-values <0.05.‬‬‬‬‬‬‬‬‬‬‬‬‬‬‬‬‬‬‬‬‬

**Fig. 1:**
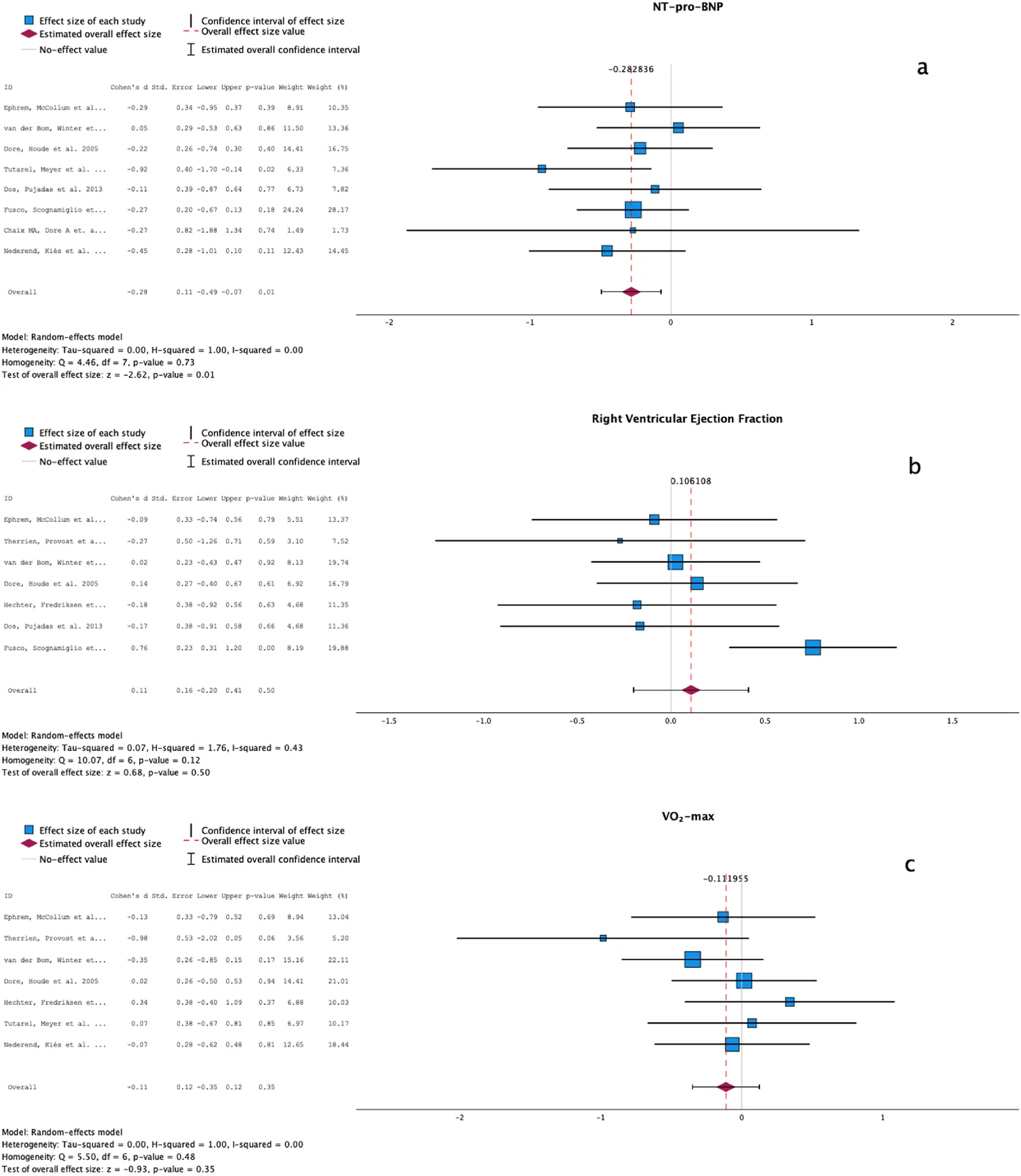
Forest plots of outcomes following RAAS inhibition in patients with sRV. **(a)** NT-pro-BNP: Pooled effect size across 8 studies demonstrating a reduction in NT-pro-BNP levels following RAAS inhibition (Cohen's d = −0.28, 95% CI: −0.49 to −0.07, p = 0.01; I^2^ = 0.0%). **(b)** RVEF: Pooled effect size across 7 studies showing a neutral effect on RVEF (Cohen's d = 0.11, 95% CI: −0.20 to 0.41, p = 0.50; I^2^ = 43.0%). **(c)** VO_2_-max: Pooled effect size across 7 studies demonstrating no change in peak oxygen uptake (Cohen's d = −0.11, 95% CI: −0.35 to 0.12, p = 0.35; I^2^ = 0.0%). Blue squares represent individual study effect sizes (Cohen's d), with square size proportional to the relative weight assigned in the random-effects model. Horizontal lines denote 95% confidence intervals (CI). The red diamond represents the overall pooled effect size and its 95% CI. The solid vertical line indicates the line of no effect (0.0), and the vertical red dashed line marks the overall effect estimate. All analyses were performed using a random-effects model. Abbreviations: CI = confidence interval; NT-pro-BNP = N-terminal pro-b-type natriuretic peptide; RAAS = renin-angiotensin-aldosterone system; RVEF = right ventricular ejection fraction; VO_2_-max = maximal oxygen uptake.

For the trial by Chaix et al. [14], individual patient NT-pro-BNP values were extracted directly from Figure 1D in the original cited study by Chaix et al. [14] (n = 3), allowing for calculation of the group sample mean and SD using standard formulas for sample variance.‬‬

Pooled percentages across the global cohort (*e.g*., overall sex distribution, anatomical proportions, and raw mortality rates) were calculated using total sample denominators from reporting studies.

## 3 Results

### 3.1 Study selection

The initial systematic literature search across the PubMed database yielded a total of 46 potentially relevant citations. After initial screening of titles and abstracts, 32 records were excluded as they clearly did not meet the predefined eligibility criteria.

Subsequently, 14 articles were retrieved and subjected to a comprehensive full-text review. Of these, 4 studies were excluded for specific reasons, including lack of relevant clinical outcome data and overlapping cohorts. Following the screening process, a total of 10 clinical studies met the inclusion criteria and were assessed in the meta-analysis.

### 3.2 Study and patient characteristics

Ten eligible studies were evaluated, comprising a pooled cohort of 325 patients with a sRV. Mean pharmacological treatment durations ranged across the cohorts from 3.5 to 37.2 months, and reference control arms were utilized in six of the ten included trials (Table 1Table 2).

**Table 1:** Overview of included studies.

	Study design	Age (mean ± SD, *years*)	Sex	Anatomy	Studied medication	Follow up time (years)
Ephrem et al., 2022	Observational	40.2 ± 6.7	M: 13 (72%)	TGA-AS: 16 ccTGA: 2	ARNI	1.10 ± 0.6
Therrien et al., 2008	RCT	26.4 ± 5.2	M: 11 (65%)	TGA-AS: 17	ARB	1.00 ± 0
van der Bom et al., 2013	RCT	33 ± 10	M: 57 (65%)	TGA-AS: 63 ccTGA: 25	ARB	3.10 ± 0.9
Dore et al., 2005	Crossover clinical trial	30.3 ± 10.9	M: 24 (83%)	TGA-AS: 21 ccTGA: 8	ARB	0.29 ± 0.2
Hechter et al., 2001	Observational	33.0 ± 13.2	M: 12 (86%)	TGA-AS: 14	ACEi	18 m (range 6- 56)
Tutarel et al., 2012	Observational	25.2 ± 3.5	M: 21 (75%)	TGA-AS: 14	ACEi	1.1 ± 0.3
Dos et al., 2013	RCT	26.4 ± 5.4	M: 16 (61.5%)	TGA-AS: 26	MRA	1.0 ± 0
Fusco et al., 2023	Observational	38 ± 12	M: 30 (60%)	TGA-AS: 33 ccTGA: 18	ARNI	1.0 ± 0
Chaix MA et al., 2024	RCT	48.5 ± 7.3	N/A	TGA-AS: 14 ccTGA: 1	ARNI	0.3 ± 0.24
Nederend et al., 2023	Observational	48.3 ± 6.9	M: 24 (60%)	TGA-AS: 28 ccTGA: 12	ARNI	2.0 ± 1.45

Baseline characteristics, study designs, anatomical distribution, and follow-up durations of the included studies. Abbreviations: ACEi, angiotensin-converting enzyme inhibitor; ARB, angiotensin receptor blocker; ARNI, angiotensin receptor-neprilysin inhibitor; ccTGA, congenitally corrected transposition of the great arteries; M, male; F, female; MRA, mineralocorticoid receptor antagonist; N/A, not applicable/available; RCT, randomized controlled trial; TGA-AS, transposition of the great arteries status-post atrial switch surgery (Mustard or Senning).

**Table 2:** Safety profiles.

Study	Studied medication	Discontinuations due to AE (n, %)	Most Common AE	SAEs & Deaths
Ephrem, McCollum et al., 2022	ARNI	0 (0%)*	None reported.	4 hospitalizations for worsening heart failure during follow-up. No deaths. *Note: 2 patients discontinued only to undergo successful heart transplantation.
Therrien, Provost et al., 2008	ARB	0 (0%)*	None reported.	None reported. *Note: 10 patients withdrew due to personal/practical reasons (pregnancy, psychological distress, lack of motivation, missed MRI).
van der Bom, Winter et al., 2013	ARB	Active: 9 (20.5%) Placebo: 5 (11.4%) (*p* = 0.38)	Fatigue (Valsartan: 4; Placebo: 2), dry cough, palpitations, thigh bruising, elevated liver enzymes, and increased arrhythmia frequency.	2 deaths (1 in Valsartan (pulmonary complications), 1 in Placebo (sudden cardiac death during HF hospitalization)). Clinical events across cohort: supraventricular arrhythmias (18%), worsening HF (13%), ventricular tachycardia (5%). 2 active-arm patients underwent cardiac surgery.
Dore, Houde et al., 2005	ARB	0 (0%)*	Mild dyspnea, fatigue, palpitations, dizziness, diarrhea, cough, and muscle pain (similar rates between active drug and placebo).	None reported. *Note: 6 patients dropped out due to non-compliance or difficulty swallowing tablets, unrelated to side effects.
Hechter, Fredriksen et al., 2001	ACEi	0 (0%)	Safe; expected therapeutic reduction of maximal systolic blood pressure during exercise (36% of patients).	None reported.
Tutarel, Meyer et al., 2012	ACEi	0 (0%)	None reported.	None reported.
Dos, Pujadas et al., 2013	MRA	2 (11.1%)*	Mild dizziness, palpitations, urinary frequency, and pruritus.	1 case of severe hyperkalemia (6.1 mmol/L) in a patient with concomitant ACE-I therapy (normalised after drug withdrawal 2 weeks early). No deaths. *Note: 1 patient discontinued due to dizziness, and 1 separate patient was withdrawn due to hyperkalemia.
Fusco, Scognamiglio et al., 2023	ARNI	2 (4%)	Symptomatic hypotension was the primary dose-limiting factor (limiting titration in 54% of patients). SVT (12%, n = 6), ventricular runs (6%, n = 3).	1 patient (2%) developed a nephrotic syndrome deemed unrelated to active drug). No deaths.
Chaix MA, Dore A, Mondésert B et al., 2024	ARNI	Trial terminated early due to safety concerns.	Syncope due to orthostatic hypotension (n = 1). Mild orthostatic hypotension, erectile dysfunction, photosensitivity, dizziness, headache, diarrhea, and insomnia.	High rate of acute heart failure exacerbations during drug-free washout periods: 71.4% (5 of 7) of the placebo-onset group and 2 patients in the active-to-washout group developed acute HF. No deaths.
Nederend, Kiés et al., 2023	ARNI	9 (22.5%)*	Orthostatic hypotension was the main reason for not reaching target dose (89% of cases). Uncontrollable thirst (n = 1).	1 death (cardiogenic shock in end-stage HF during titration). 2 cases of worsening heart failure. *Note: 5 patients discontinued during titration, and 4 additional patients discontinued during follow-up (2 due to heart transplant, 1 renal decline, 1 dizziness/fatigue).

Summary of safety profiles, adverse events, treatment discontinuations, and serious clinical events across the included studies. Abbreviations: ACEi, angiotensin-converting enzyme inhibitor; AE, adverse event; ARB, angiotensin receptor blocker; ARNI, angiotensin receptor-neprilysin inhibitor; HF, heart failure; MRA, mineralocorticoid receptor antagonist; MRI, magnetic resonance imaging; SAE, serious adverse event; SVT, supraventricular tachycardia; TV, tricuspid valve.

In terms of baseline congenital anatomy, the majority of the analyzed population consisted of patients with d-TGA who had undergone a surgical atrial switch procedure (Mustard or Senning), accounting for 259 patients (79.7%). The remaining 66 patients (20.3%) presented with ccTGA (Table 1).

A male predominance was observed across the global study population. Characterization of participant sex was reported in nine out of the ten studies, among the 310 patients with available demographic data, 208 (67.1%) were male and 102 (32.9%) were female (Table 1). Notably, the brief report by Chaix et al. (2024) was the only study that did not provide a baseline sex breakdown for its 15 enrolled subjects. The pooled cohorts predominantly comprised young to middle-aged adults, with mean baseline cohort ages ranging from 25.2 ± 3.5 years to 48.5 ± 7.3 years (Table 1).

### 3.3 NT-pro-BNP

A pooled analysis of eight studies was performed to evaluate the changes in NT-pro-BNP levels from baseline to follow-up after RAAS inhibition. Among the individual trials, a trend toward a reduction in NT-proBNP was observed, although the magnitude and statistical significance varied. Using a random-effects model, the overall effect size demonstrated a reduction in NT-pro-BNP (SMD: −0.28, 95% CI: −0.49 to −0.07, *p* = 0.01) (Fig. 1a). One study demonstrated a decrease in NT-pro-BNP on their own (SMD: −0.92, 95% CI: −1.70 to −0.14, p = 0.02) [19] (Fig. 1a) (Table 3).

**Table 3:** Clinical efficacy outcomes.

	NT-pro-BNP	VO_2_-max	RVEF (%)
Ephrem, McCollum et al., 2022	B: 681 ± 1807 FU: 304 ± 315	B: 18.0 ± 4.4 FU: 17.4 ± 4.7	B: 32.4 ± 7.6 FU: 31.6 ± 10.1
Therrien, Provost et al., 2008	N/A	B: 24.9 ± 3.2 FU: 22.2 ± 2.2	B: 43.8 ± 7.1 FU: 40.9 ± 13.3
van der Bom, Winter et al., 2013	B: 271 ± 241 FU: 281 ± 130	**B: 27.5 ± 7.9** **FU: 24.8 ± 7.6**	B: 34.9 ± 7.4 FU: 35.1 ± 9.6
Dore, Houde et al., 2005	B: 258 ± 243 FU: 201 ± 268	B: 29.8 ± 5.6 FU: 29.9 ± 5.4	B: 41.6 ± 9.3 FU: 43.1 ± 12.2
Hechter, Fredriksen et al., 2001	N/A	B: 16.7 ± 5.1 FU: 18.5 ± 5.4	B: 47 ± 11 FU: 45 ± 11
Tutarel, Meyer et al., 2012	**B: 242 ± 105** **FU: 151 ± 93**	B: 25.6 ± 4.8 FU: 26 ± 6	N/A
Dos, Pujadas et al., 2013	B: 172 ± 203 FU: 152 ± 65	N/A	B: 54.6 ± 8.4 FU: 53 ± 10.7
Fusco, Scognamiglio et al., 2023	B: 239 ± 204 FU: 181 ± 227	N/A	**B: 35.6 ± 8.1** **FU: 41.5 ± 7.5**
Chaix MA, Dore A, Mondésert B et al., 2024	B: 688 ± 383 FU: 591 ± 331	N/A	N/A
Nederend, Kiés et al., 2023	**B: 692 ± 588** **FU: 449 ± 418**	B: 17.9 ± 4.73 FU: 17.6 ± 4.02	N/A

Baseline and follow-up clinical efficacy outcomes across the included studies, categorized by neurohormonal biomarkers, exercise capacity, and right ventricular systolic function. Values are expressed as mean ± SD. Bold values indicate statistically significant changes over the study period within that individual trial as reported by the authors. Abbreviations: B, baseline; FU, follow-up; N/A, not applicable/available; NT-pro-BNP, N-terminal pro-B-type natriuretic peptide; RVEF, right ventricular ejection fraction; VO_2_-max, maximal oxygen uptake.

### 3.4 Right ventricular ejection function (RVEF)

A pooled analysis of seven studies was conducted to evaluate the impact of RAAS inhibition on RVEF. Utilizing a random-effects model, the overall effect size demonstrated no difference regarding RVEF following treatment (Standardized Mean Difference (SMD): 0.11, 95% CI: −0.20 to 0.41, p = 0.5).

One study demonstrated improvement in ventricular function (SMD: 0.76, 95% CI: 0.31 to 1.20) [15]. The remaining six studies demonstrated neutral effects, with confidence intervals spanning across the line of no effect (Fig. 1b) (Table 3).

### 3.5 VO_2_-max

VO_2_-max was evaluated across seven studies. The pooled analysis (random-effects model) revealed no change in VO_2_-max from baseline to follow up, (SMD: −0.11, 95% CI: −0.35 to 0.12, *p* = 0.35). Furthermore, no individual study showed any difference either (Fig. 1c) (Table 3).

### 3.6 Mortality and other adverse events

The overall pooled treatment mortality was low across the analyzed studies. Among the 261 treated patients, two deaths occurred during the active trial periods, corresponding to a raw pooled mortality rate of 0.77%. One death, due to further described pulmonary complications, occurred in the active treatment arm of one of the trials having ARB as the studied medication [20]. The second death, due to cardiogenic shock during the titration phase, was reported in the prospective cohort, having ARNI as the studied medication [17]. Additionally, one death (sudden cardiac death) was recorded in the placebo arm (n = 44), representing a placebo-group mortality rate of 2.3% in that trial [20].

When accounting for differences in follow-up durations using person-years of exposure, a long-term observational follow-up study of the largest randomized cohort demonstrated a mortality rate of 7.3 deaths per 1000 person-years [21]. In comparison, the mortality rate in the shorter-term cohort was 15.6 deaths per 1000 person-years [17]. The pooled incidence rate of the treated group was 5.6 deaths per 1000 person-years.

Safety profiles and treatment tolerability varied across different pharmacological classes (Table 2). Adverse events leading to premature treatment discontinuation or clinical concern were documented in 6 out of the 10 studies.

Trials investigating fixed doses of ACE-inhibitors or ARBs reported high tolerability with 0% discontinuation rates due to side effects [19,[22], [23], [24]]. Conversely, trials utilizing active dose-titration protocols reported discontinuation rates ranging from 4% [15] (due to symptomatic hypotension) to 22.5% [17] (due to a combination of hypotension, heart failure progression, and renal decline).

In the crossover trial, that required a drug-free washout period, acute heart failure hospitalizations occurred in 5 out of 7 of the placebo-onset group and in 2 patients out of 8 in the active-to-washout group, leading to the early termination of the study [14].

## 4 Discussion

The pooled data from several clinical investigations of different designs show that RAAS inhibition in sRV is safe and has positive effects on NT-pro-BNP whereas there was no change in ejection fraction or peak VO_2_. Together with extrapolated data from acquired left ventricular failure [[25], [26], [27], [28], [29]], the results from this meta-analysis support the judicious use of RAAS inhibition in sRV failure. The prevalence of side effects was low and of the expected type. The mortality must be considered in the context of severe congenital heart disease and was, relative to acquired heart failure, low [26,28]. Still, the prophylactic use of RAAS inhibition in patients with sRV or low grade sRV dysfunction remains open.

NT-pro-BNP was the only variable that showed positive effects after initiation of RAAS blockade that is in line with major trials on acquired left ventricular failure. Given the relative short duration of treatment across the included studies and the major anatomical constraints of the sRV, this indicates a positive effect on the overloaded sRV. There was no detectable effect on ejection fraction nor peak VO_2_, analogous to major HF clinical trials [25,27,30]. It must also be considered that our pooled study population is relatively small, especially when compared to the major clinical trials on acquired heart failure [[25], [26], [27],^29^,^30^].

In our meta-analysis, mortality was low and occurred in three subjects, one of which was in a placebo arm. In neither of the cases, the tested treatment was obviously related to the deaths. Conversely, high rates of acute heart failure exacerbations were documented during the drug-free washout periods, illustrating the potential danger of abruptly withdrawing neurohormonal blockade in this vulnerable population [14]. Ultimately, the trials and very likely also the meta-analysis is much too small to detect a difference in mortality between placebo and active treatment as well as for other major adverse cardiovascular events.

In patients with sRV where prophylactic treatment may be an option when the defect is diagnosed long time before any symptoms occur and even before there are signs of ventricular dysfunction, there is presently no guiding trials. In this meta-analysis, at least a part of the patients did not have overt heart failure but the follow up is probably too short to evaluate the long-term effect. Nevertheless, the use of ACEi and ARB in arterial hypertension support the general long-term safety with these drugs [31].

The 2020 ESC ACHD guideline states that in patients with sRV, there is “no proven benefit from heart failure medical therapy” for outcomes, although “classical” heart failure drugs or ARBs may provide some benefit in more symptomatic patients, also stating “there are currently only a few small studies looking at the new drug sacubitril/valsartan” “however, no recommendation can be made at this moment.“. The 2021 ESC Heart Failure guideline adds that extrapolation from standard HF trials to ACHD is not always appropriate, especially in patients with sRV, and that the few available data are inconclusive and mostly based on small cohorts/clinical experience. This meta-analysis provides further quantitative support for this cautious approach. The population of patients with atrial switch operations constantly decrease as the primary reconstruction since decades is arterial switch that puts the left ventricle in the systemic position. Patients with ccTGA will in a future need mediation. The relative low incidence of ccTGA, the high frequence of late diagnosis and the need for a long observation time make RCTs difficult to perform. The present data however supports the use of RAAS-inhibition in patients with a sRV, albeit without hard cardiovascular endpoints.

### 4.1 Conclusion

In patients with sRV, RAAS inhibition appears safe, well-tolerated, and associated with a reduction in NT-pro-BNP levels, suggesting a favourable hemodynamic offloading effect on the strained ventricle. However, these pharmacological interventions do not yield short-term improvements in RVEF or peak VO_2_. Furthermore, the low overall treatment mortality and the high rate of heart failure exacerbations observed during drug withdrawal underscore the safety of therapy as well as the potential risks of abrupt discontinuation. Together, these findings provide quantitative support for current international guidelines, endorsing the judicious, individualized use of RAAS inhibition in sRV.

## CRediT authorship contribution statement

**Yousef Baroudi:** Conceptualization, Data curation, Formal analysis, Investigation, Methodology, Validation, Visualization, Writing – original draft, Writing – review & editing. **Bengt Johansson:** Conceptualization, Data curation, Funding acquisition, Investigation, Methodology, Resources, Supervision, Validation, Writing – original draft, Writing – review & editing.

## Declaration of generative AI and AI-assisted technologies in the manuscript preparation process

During the preparation of this work the authors used Google Gemini for language editing. After using this tool, the authors reviewed and edited the content as needed and takes full responsibility for the content of the published article.

## Funding

The Swedish Heart and Lung Foundation 20230503, 20200493, 20190525.

County of Västerbotten

10.13039/501100004885Umeå University

Visare Norr

Norrländska hjärtfonden

Hjärtebarnsfonden

## Declaration of competing interests

The authors declare that they have no known competing financial interests or personal relationships that could have appeared to influence the work reported in this paper.
