# Supplementary material for: Meta-analysis of RAAS inhibition in patients with a systemic right ventricle
Source: Int J Cardiol Congenit Heart Dis. 2026 Aug 13;25:100705. doi: 10.1016/j.ijcchd.2026.100705 (PMC13551887; doi:10.1016/j.ijcchd.2026.100705)
Supplement: Supplementary file 1 [file mmc1.docx]

# Supplementary file

## The complete search strategy

This gave all the articles: ("systemic right ventricle"[Title/Abstract] OR "transposition of the great arteries"[MeSH Terms] OR "atrial switch"[Title/Abstract] OR "Mustard"[Title/Abstract] OR "Senning"[Title/Abstract] OR "congenitally corrected transposition"[Title/Abstract] OR "ccTGA"[Title/Abstract] OR "d-TGA"[Title/Abstract]) AND ("angiotensin-converting enzyme inhibitors"[MeSH Terms] OR "angiotensin receptor antagonists"[MeSH Terms] OR "mineralocorticoid receptor antagonists"[MeSH Terms] OR "neprilysin"[MeSH Terms] OR "sacubitril valsartan"[Title/Abstract] OR "ACEi"[Title/Abstract] OR "ARB"[Title/Abstract] OR "ARNI"[Title/Abstract] OR "RAAS blockade"[Title/Abstract])

## Data extraction for Chaix et al. (2024)

Extracted NT-proBNP data for Chaix et al. (2024) digitally extracted from Figure 1D of the original publication (n = 3‬‬).

Individual Baseline values (pg/mL): Patient 1: 250, Patient 2: 780, Patient 3: 1034; Mean ± SD: 688.0 ± 383.2 pg/mL

Individual Follow-up values (pg/mL): Patient 1: 210, Patient 2: 680, Patient 3: 882; Mean ± SD: 590.7 ± 330.7 pg/mL
